# *Chryseobacterium gleum* bacteraemia: first reported cases from Qatar

**DOI:** 10.1016/j.nmni.2021.100869

**Published:** 2021-03-22

**Authors:** M. Ali, H. Alsoub

**Affiliations:** Department of Infectious Disease, Medicine Department, Hamad Medical Corporation, Doha, Qatar

**Keywords:** Antibiotic resistant, bloodstream infection, *Chryseobacterium gleum*, hospital acquired infection, infection control

## Abstract

*Chryseobacterium gleum* is a Gram-negative aerobic bacillus. It commonly colonizes mechanical devices, causing device-associated infections like central line–associated bloodstream infection and ventilator-associated pneumonia. We describe two cases of *C. gleum* bacteraemia in patients admitted to our intensive care unit in Qatar, one of which resulted in death. Long hospital stays and indwelling devices are risk factors for *C. gleum* bacteraemia. Because *C. gleum* is inherently resistant to β-lactam antibiotics, rapid identification and antimicrobial susceptibility testing are essential for guiding therapy.

## Introduction

*Chryseobacterium gleum* (formerly *Flavobacterium gleum*) [1], is a Gram-negative, nonfermenting, catalase-positive and indole-positive aerobic bacillus [2]. It commonly found on moist hospital surfaces, like washbasins and dressing trolleys [3]. Inadequate infection control makes it a risk for hospital-acquired infection and its transmission between patients [4] because they can colonize mechanical devices, causing device-associated infections like central line–associated bloodstream infection (CLABSI) and ventilator-associated pneumonia [3,4].

*Chryseobacterium gleum* is infrequently isolated and has been reported in several countries including India, Hungary, Croatia, Qatar, Taiwan and Saudi Arabia [[5], [6], [7], [8], [9], [10]]. *Chryseobacterium gleum* spp. are resistant to several antibiotics such as aminoglycosides, chloramphenicol, tetracycline, clindamycin, teicoplanin and erythromycin [11,12]. In addition, these strains chromosomally encode class A carbapenemases and class B metallo-β-lactamases which confer resistance to all β-lactams [13].

Here we describe what is to our knowledge the first outbreak of *C. gleum* infection in Qatar. Ethical approval was received from Hamad Medical Corporation's Medical Research Council.

## Case report

### Case 1

A 75-year-old woman with a history of end-stage renal failure undergoing haemodialysis through fistula, diabetes mellitus and hypertension was admitted to our hospital complaining of acute abdominal pain. She was found to have acute mesenteric artery thrombosis and multiple splenic hypodensities by computed tomographic scan. She had concurrent *Klebsiella pneumonia* bacteraemia managed with piperacillin/tazobactam. Her course was complicated by a splenic abscess, which was initially treated with the antibiotic piperacillin/tazobactam; however, she experienced cardiac arrest, was resuscitated and was then moved to the intensive care unit (ICU). During her stay, splenic abscess drainage was done by the intervention radiologist, and culture grew *Parabacteroides distasonis* which was sensitive to metronidazole and resistant to amoxicillin/clavulanic acid; it was treated with metronidazole. Five days later, the patient experienced septic shock. Blood culture of a peripheral line grew *Chryseobacterium gleum.* It was sensitive only to ciprofloxacin and levofloxacin, and was resistant to all other antibiotics including piperacillin/tazobactam, meropenem, colistin, tigecycline, amikacin and cotrimoxazole. The patient was diagnosed with a CLABSI, so the line was removed and the patient was treated with ciprofloxacin (400 mg provided intravenously daily for 2 weeks). The bacteraemia cleared after 1 week. However, the patient had another cardiac arrest and died 2 weeks after her new infection was detected.

## Case 2

A 73-year-old man was admitted to our hospital in December 2018 with septic shock that had resulted from a perianal abscess complicated with Fournier gangrene which was treated with antibiotics. His hospital course was also complicated by *Clostridium difficile* infection. In January 2019, during his ICU admission, he again experienced septic shock. Blood culture from central and peripheral lines showed *C. gleum* and *Acinetobacter baumannii* bacteraemia. The profile for *C. gleum* showed sensitivity to levofloxacin, amikacin and cotrimoxazole and intermediate sensitivity to ciprofloxacin. The patient was treated with levofloxacin (750 mg provided intravenously daily for 2 weeks) and piperacillin/tazobactam (to cover *Acinetobacter*), and the central line was removed. The patient's infection was cured.

## Discussion

*Chryseobacterium gleum* is an unusual human pathogen that has been reported as a cause for hospital-acquired infection. Most of publications available in the literature refer to long hospital stays or indwelling devices. The organism has also been reported in cystic fibrosis patients [14]. One study in Qatar in 2015 identified *C. gleum* from a respiratory sample from a patient with cystic fibrosis [6].

A 2015 report from Croatia described a case of *C. gleum* infection in a patient with malnutrition and hepatic lesion [8]. A case published in 2016 from Saudi Arabia reported *C. gleum* pneumonia in a 6-month-old baby with nephrotic syndrome [10]. Reports of *C. gleum* infection are summarized in Table 1. Our patients had line-related bacteraemia with no other source of infection. We performed an outbreak investigation because both cases happened simultaneously; however, their MIC profiles showed some differences (Table 2). This organism is capable of producing Ambler class B carbapenem-hydrolyzing β-lactamase, which might cause treatment failure when β-lactam antibiotics are used as a first-line treatment. The organism shows different sensitivity profile to fluoroquinolones and cotrimoxazole.

**Table 1:** Summary of reports of isolation of *Chryseobacterium gleum*

Study	Year	Country	Patient description	Sample	Therapy	Response
Lambiase [14]	2007	Italy	2 patients with cystic fibrosis	Respiratory	NA	
Lo [7]	2014	Taiwan	3 male and 1 female hospitalized patients	Respiratory	NA	
Virok [5]	2014	Hungary	3 neonates with early-onset infection	Respiratory	Ciprofloxacin	Yes
Brkic [8]	2015	Croatia	1 female patient with severe malnutrition and hepatic lesion	Bacteraemia	Piperacillin/tazobactam	Yes
Abdalhamid [10]	2016	Saudi Arabia	1 infant with nephrotic syndrome	Respiratory	Levofloxacin	Yes
Rawat [15]	2017	India	1 infant with chronic granulomatous disease	Respiratory	Piperacillin/tazobactam and cotrimoxazole	Yes
Jain [16]	2017	India	1 male patient with tentorial bleed after road traffic accident	Respiratory + bacteraemia	Levofloxacin	Yes
Arouna [17]	2017	Senegal	1 male patient after prostatectomy	Urine	Ciprofloxacin	Yes
Singhal [18]	2017	India	1 male patient with COPD and sepsis	Bacteraemia	Levofloxacin	Yes
This study, patient 1	2019	Qatar	1 female patient with septic shock	Bacteraemia	Ciprofloxacin	Died
This study, patient 2	2019	Qatar	1 male patient with renal failure and septic shock	Bacteraemia	Levofloxacin	Yes

Abbreviations: COPD, chronic obstructive pulmonary disease; NA, not applicable.

**Table 2:** MICs of various antibiotics for *Chryseobacterium gleum*

Antimicrobial agent	MIC (μg/mL) (interpretation) for:	
	Patient 1	Patient 2
Meropenem	32 (R)	>8 (R)
Ciprofloxacin	0.75 (S)	1.5 (I)
Cotrimoxazole	6 (R)	1/19 (S)
Cefepime	1.5 (S)	>16 (R)
Piperacillin/tazobactam	256 (R)	>64/4 (R)
Ceftazidime/avibactam	256 (R)	>16 (R)
levofloxacin	0.5 (S)	2 (S)
Amikacin	256 (R)	16 (S)
Colistin	256 (R)	>4 (R)

Abbreviations: I, intermediate; R, resistant; S, sensitive.

## Conclusion

Critically ill patients in ICUs are at risk of healthcare-associated infection due to the emerging pathogen *C. gleum*. Long hospital stays and the presence of indwelling devices are risk factors for *C. gleum* bacteraemia. Proper infection control practices and outbreak investigations are essential to prevent the spread of infections related to this organism. Because *C. gleum* is inherently resistant to β-lactam antibiotics, rapid identification and antimicrobial susceptibility testing are essential for guiding therapy.

## Conflict of interest

None declared.
